# Novelty Experience in Prolonged Interaction: A Qualitative Study of Socially-Isolated College Students’ In-Home Use of a Robot Companion Animal

**DOI:** 10.3389/frobt.2022.733078

**Published:** 2022-03-11

**Authors:** Bryan Abendschein, Autumn Edwards, Chad Edwards

**Affiliations:** Communication and Social Robotics Labs, School of Communication, Western Michigan University, Kalamazoo, MI, United States

**Keywords:** novelty, social robot, human-robot interaction, dialectics, user experience, COVID-19, well-being

## Abstract

Social distancing policies such as limits on public gatherings and contact with others were utilized around the world to slow the spread of COVID-19. Yet, decreased social interactions may also threaten people’s well-being. In this project, we sought to understand novelty-relevant experiences surrounding in-home companion robot pets for adults that were living in some degree of social isolation due to the COVID-19 pandemic. After 6-weeks of participants living with the robot companion, we conducted semi-structured interviews (*N* = 9) and six themes emerged from our iterative analysis (expectations versus reality, ontological comparisons, interactions, third-party influence, identity, and comfort). Findings suggest that novelty is a complex phenomenon consisting of various elements (i.e., imagined novelty, technology novelty, and relational novelty). Each component influences the user’s experience. Our findings also suggest that our understanding of novelty as a nonlinear resource may hold important implications for how we view human-robot relationships beyond initial encounters.

## 1 Introduction

The COVID-19 pandemic continues to be a stressful event for college students (Son et al., 2020). During this time, researchers found that around 48% of college students were experiencing moderate-to-severe levels of depression, and 40% were showing heightened signs of anxiety (Wang et al., 2017a). In addition to everyday stressors like final exams (Barker et al., 2016), COVID-19 health precautions significantly increased mental health issues for students (Lederer et al., 2020). In response to the pandemic, many United States colleges and universities shifted to online education, sent students back home, and had to shut down social events. Students lost college jobs and were forced to make life-changing decisions while attending virtual classes with related assignments and exams. As such, “the COVID-19 pandemic has exacerbated college students’ known mental health risk factors and other health concerns while simultaneously imperiling students’ academic outcomes, putting their future prospects dependent on college retention in jeopardy” (Lederer et al., 2020, p. 15).

Social distancing policies such as the closure of public spaces and limited contact with others continue to be in place to slow the spread of COVID-19. Yet, decreased social interactions threaten people’s well-being and mental health by increasing feelings of anxiety, depression, and loneliness (Usher et al., 2020). This project seeks to investigate novelty-relevant experiences surrounding the adoption of robot pets by adults during the COVID-19 pandemic. In doing so, we hope to understand the effects of living with a robotic animal companion for 6 weeks during a period when many people experienced the acute impact of social isolation.

Social isolation, or limited opportunities to connect with others, was an issue facing many adult populations even before the pandemic (Simone et al., 2021), but COVID-19 introduced new challenges. College students, for example, who enrolled in face-to-face classes found those courses moving online. Additionally, many universities announced they would be temporarily closing, or limiting, on-campus housing options and halting social gatherings due to the pandemic. Apart from recent events, in-home robot companion animals have been intentionally designed and marketed towards the provision of care to socially isolated people in the past (Coghlan, 2021). In the following sections, we will address college student well-being, stress and anxiety, and robot companions. We will also explore the current research on novelty effects in HRI.

## 2 College Student Stress and Well-Being

College students regularly experience moderate stress levels (Pierceall and Keim, 2007), and stress reduction is tied to life satisfaction and well-being (Delgado et al., 2018). In this study, we conceptually align well-being with comfort and opportunities for social interaction. Due to family separation, career development, and relationship exploration, however, the period of emerging adulthood (i.e., 18–25 years old) may be accompanied by increased stress (Arnett, 2000; Coccia and Darling, 2016). It is common to have elevated depression and overall anxiety during psychological development (Gilbert et al., 2009). Social support and comforting measures can be employed both on the individual and the institutional levels. Interactions that foster comfort can help boost performance Nicpon et al. (2006), reduce anxiety (Lepore et al., 1993), decrease the feelings of loneliness (Mattanah et al., 2010), and increase physical health (Brashers et al., 2004). For this study, we define social support/comforting behaviors as an interpersonal resource provided through others (Albrecht et al., 1992) and directly related to a sense of well-being.

At the institutional level, in normal years, colleges and universities offer ways to help students negotiate depression, anxiety, and well-being. Some institutions of higher education have used mindfulness training (Byrne et al., 2013), yoga (Malathi and Damodaran, 1999), and even pet therapies (Daltry and Mehr, 2015; Delgado et al., 2018). Research has demonstrated that therapy dogs can reduce perceived student stress and increase general well-being (Barker et al., 2016), as college students often turn to animals for comfort (Kurdek, 2009). These studies have demonstrated large effect sizes for stress reduction with college students. However, during the COVID-19 pandemic, most of these support programs were not available. Because individuals may not be available to offer support and comfort physically, many college students have turned to their pets. However, at the university, the use of animals is not without limitations. People could have negative health effects due to allergies (Perkins, 2020), animals need to be trained for this type of support (Daltry and Mehr, 2015), and there is the potential for harm to the therapy animals (Jalongo and McDevitt, 2015). Some have argued that robot pets might provide a safer alternative that can achieve similar effects. In the following section, we will explore the use of robots to provide socially supportive contact, defined as supportive interactions without the anticipation of reciprocity (Edwards A. et al., 2020).

### 2.1 Robot Pets

A long line of research using the computers are social actors paradigm has argued that people will treat and interact with computers in similar ways to how they treat and interact with other people (Reeves and Nass, 1996; Nass and Moon, 2000). Additionally, research has shown that humans will apply similar social scripts to robots (Spence et al., 2014; De Graaf et al., 2015; Edwards, 2018; Edwards et al., 2019; Edwards A. et al., 2020; Edwards C. et al., 2020; Gambino et al., 2020; Abendschein et al., 2021b). Previous literature suggests that robot pet therapies will offer socially supportive contact and stress reduction for college students that may relate to the social scripts we associate with other companion animals.

Zoomorphic robots have been utilized among older populations with much success (Takayanagi et al., 2014; Lazar et al., 2016) and have been specifically studied with older adults who have diminished cognitive function (Heerink et al., 2010). For younger people, Paro the Seal has been found to reduce pain perceptions (Geva et al., 2020). In fact, researchers showed that the positive effects for their older adult sample were not due to short-term novelty effects but rather the interaction produced around the robot pet (Šabanović et al., 2013). Our previous research involving college students demonstrated a significant decrease in feelings of stress after a brief exposure to robot animal companions during final exams week. Students rated themselves significantly happier, more relaxed, less exhausted, and less bored following short unstructured interactions. Effect sizes ranged from small (0.20) to large (0.70), with the most considerable effects on levels of stress and relaxation (Edwards A. et al., 2020). Although this study provided a brief exposure of 15 min or less, participants reported this new experience as significant and meaningful. The following section outlines novelty effects in human-robot interactions as they relate to this 6-week in-home user study of college students during the COVID-19 pandemic.

### 2.2 Novelty Effects

The heightened awareness that people feel when encountering a new device may influence their initial impressions but also as a hallmark of that experience (Smedegaard, 2019). “Novelty arises as a feature of experience, when we encounter something that we cannot make sense of solely in terms of what we already know” (Smedegaard, 2019, p. 413). In other words, novelty is not a property of technologies but rather a unique aspect of people’s encounters and experiences with technologies. Additionally, a long line of interpersonal communication research demonstrates that people also face, desire, and negotiate the experience of novelty in their relationships with other human beings. In the context of human social relationships, novelty has been theorized as a dialectical tension; something desired and valued, but existing in opposition to an equally valid and often competing need for familiarity and predictability (Graham, 2003; Baxter, 2006). In this sense, the practice of relating to another necessarily involves oscillating efforts between one’s desire to renew novelty and the heightened interest and excitement it entails and to pursue sameness and stability. Because the experience of novelty is dynamic, it is important to understand how it is felt, understood, and discussed.

Often labeled “novelty effects,” these experiences of newness and excitement surrounding interactions with technologies can provide an important source of information for understanding HRI by complicating an individual’s perceptions and shaping the user’s experience with social robots (Smedegaard, 2019). In part, the rareness of social robots could lead to feelings of novelty in short-term interactions. For example, one study asked participants to rate their uneasiness while looking at “hard-to-categorize” stimuli (i.e., human faces transposed over cartoon/doll faces), findings suggest that those who were less comfortable with novel experiences in their lives, in general, felt more unease while looking at the faces when compared to other participants. One’s aversion to novelty could make interactions with social robots more uncomfortable for the individual. In other words, novelty effects are something to avoid or get past before the real impressions are seen (Sasaki et al., 2017).

Recent scholarship, however, has argued that we need to explore the novelty of HRI beyond the laboratory and move inquiries into the real world with longer-term studies that examine user experiences in their homes (de Graaf et al., 2018; Smedegaard, 2019). “It is important for researchers and designers of social robots to focus not only on the short-term studies, but also on long-term studies. Short-term studies may be insufficient to elicit key aspects of robots’ social behaviors that participants will identify as important” (Koay et al., 2007, p. 569). These calls have gone largely unaddressed.

However, there have been some notable exceptions. Using a 6-month in-home study, researchers demonstrated that long-term acceptance was linked to ongoing experiences with the robot over time (de Graaf et al., 2018). In another 6-month study examining the implementation of robot animals for older adults with dementia, researchers found that novelty effects were largely unwarranted and that less sophisticated, more affordable robots could be used as an alternative to more expensive, complicated robotic systems (Bradwell et al., 2020). In fact, “Studying long-term use within people’s natural environments, such as domestic environments, can provide practical insights into the continuous use of and user experiences with these systems because these environments are stable and controllable for users” (de Graaf et al., 2018, p. 2583). These studies are important for understanding how novelty may be a dynamic feature of HRI, a challenge to the prevailing assumptions about novelty.

HRI researchers have argued that while observed behaviors may change over time, they speculate those changes are due to novelty or unreasonable expectations (Kanda et al., 2004). However, these arguments often reduce novelty effects to “noise in the need of reduction” (Smedegaard, 2019, p. 412). In other words, some view novelty as dangerous to “the validity of a given study,” which introduces the risk of “confounding the generated data” (Smedegaard, 2019, p. 412). This view does not encompass the full perspective of novelty, but the prevailing position towards novelty effects in HRI seems to be that novelty is something that declines and then “wears off” over time (Leite et al., 2009, 2013; Saad et al., 2019) and that novelty decreases in linear, inverse proportion to feelings of familiarity.

Novelty is a feature of interactive experiences that may differ in intensity and change over time. It may also be thought of as one endpoint on a continuum with familiarity on the other end. (Smedegaard, 2019). Previous research has demonstrated that older adults appreciated aspects of a social robot over time as they learned more about its various capabilities (De Graaf et al., 2015). Novelty, it seems, is not simply confounding noise, but rather a crucial part of the user’s ongoing experience. Additionally, novelty effects may be “valuable sources of information” vital to understanding HRI and human-robot relationships (Smedegaard, 2019, p. 418). During both initial and prolonged exposure, novelty effects may also follow user expectations and behaviors (Rosenthal and Carter, 2021). People may experience less novelty if a social robot meets predetermined expectations. Additionally, novel interactions with social robots may contribute to anthropomorphism and viewing the robot as a viable interaction partner (Smedegaard, 2019). Their social capabilities may be convincing, but may also expose gaps in the user’s experience that influence novelty effects. However, when viewed in this way, novelty effects are not issues to be overcome but rather an opportunity to explore how interactions unfold and relationships develop between robots and humans. Toward that goal, “[I]f social robots are to be successfully introduced into people’s homes, we must understand the underlying reasons whereupon potential users decide to accept these robots and invite them into their domestic environments” (De Graaf and Allouch, 2013, p. 1476). In light of this idea and to better understand novelty as experiential, we offer the following research question to explore these ideas in a 6-week user study of robot pets.

RQ: How, if at all, did the robot kitten influence people’s experience with novelty and well-being over 6 weeks?

## 3 Materials and Methods

### 3.1 Participants

To examine the proposed research question, we analyzed interview data from nine college students enrolled at a large United States Midwestern research university (3 = women, 6 = men). The current data set is from a larger investigation (*n* = 17) that included measures not examined as part of this study. From that convenience sample of 17 individuals, nine participants were able to engage in an interview within the timeframe alloted for data collection, comprising the availability sample for this study. The average age of the sample was 22.11 years (range: 19–30), with seven participants identifying as white, one as multi-racial, and one as Asian Pacific Islander. None of our participants had previously owned a robot and only one reported prior interaction with a social robot. All participants attended college classes using virtual platforms due to the state mandates regarding higher education during the COVID-19 pandemic. Specifically, eight participants had exclusively online courses and one had a mix of online and face-to-face courses. The pandemic-related shift to online learning was difficult for many students and contributed to a sense of isolation by limiting opportunities for casual engagement with their peers (Elmer et al., 2020).

### 3.2 Hardware

**FIGURE 1:**
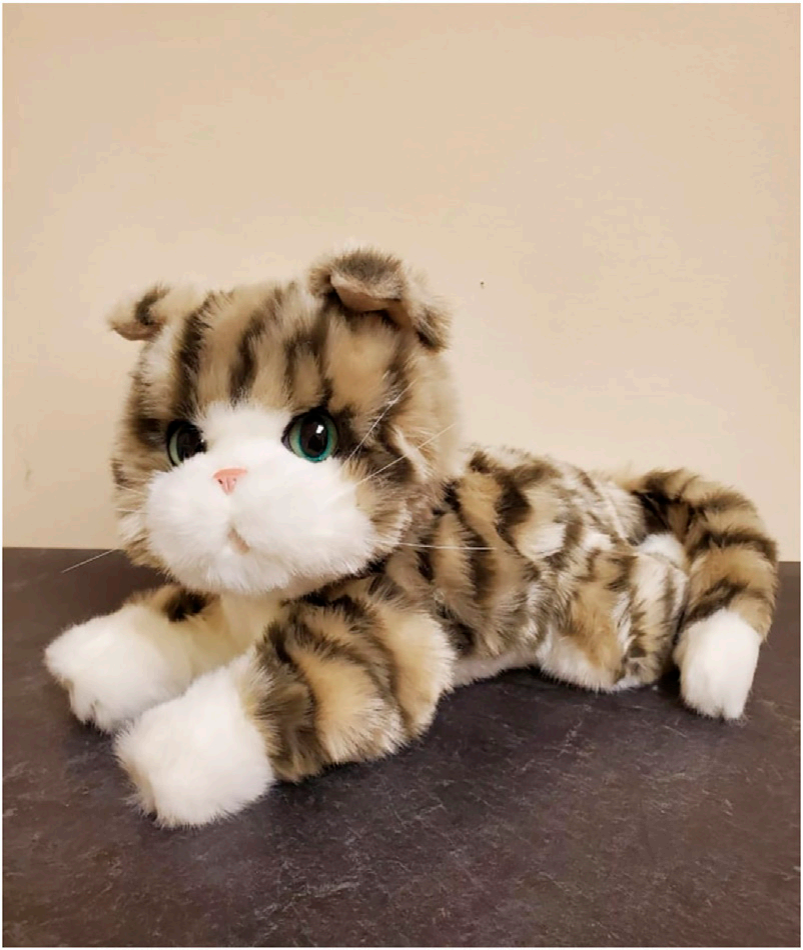
Joy for All robot kitten. The robot kitten has soft faux-fur, purrs (vibrates) using a technology the company refers to as VibraPurr, and kneads (i.e., moves its front paws up and down). The robot kitten weighs about 1 pound and takes three AA batteries.

The Joy for All Companion Pet we used for this study was a robot kitten that included a touch sensor, soft fur, and the ability to produce cat-like movements/noises (Figure 1). Specifically, the robot kitten contains a sensor plate under the fur on its back. When people touch the back of the robot kitten it purrs (i.e., vibrates gently), meows, and moves its front paws (i.e., kneading). The sounds and motions stop after a few moments unless the user continues touching the robot kitten’s back. Beyond the sensor on its back, the robot kitten does not have any learning capabilities or other means to detect the presence of users. We chose this particular robot for the study due to its simplicity since users would be unboxing it themselves and would not receive any explanation on how to use the machine.

### 3.3 Procedures

After receiving IRB approval, we invited students currently enrolled in communication courses to participate in a 6-week study examining their interactions with the robot kitten. Each participant was given a robot kitten for taking part in the study (valued at $65.00 USD). The current study examined participants’ overall thoughts about this experience using in-depth, qualitative interviews after 6 weeks. Additionally, participants took weekly surveys about their interactions with the robot, stress levels, and loneliness, but we did not analyze those measures for this study. Prior to beginning the study, participants provided informed consent using an IRB-approved consent document that outlined data collection procedures and safety protocols for participants (e.g., secure data storage, de-identified data files, and researcher contact information). Due to safety issues of face-to-face interviews related to the COVID-19 pandemic, and because the university was still holding the majority of its classes online due to social distancing protocols, we recorded each interview using a web-based meeting platform (WebEx) that generated a rough, downloadable transcript for each interview. The lead author, who is trained in qualitative interviewing and working with diverse groups of participants, conducted each interview then cleaned and checked the transcripts for accuracy. After seven interviews to examine people’s experiences with the robot kitten for this use-case study, the lead author reported reaching saturation, “when no new or relevant data seem to emerge” (Tracy, 2020, p. 227). “Typically six to seven interviews will capture the majority of themes in a homogenous sample” (Guest et al., 2020, p. 13). The lead author conducted two more interviews to collect the data from all nine available participants. After consultation as a team, we concluded that we achieved saturation for this study. This procedure aligns with qualitative methodologists’ recommendations for in-depth interviews (Guest et al., 2020; Tracy, 2020).

To begin the 6-week study, we first emailed potential participants the link to a survey with the consent document that asked participants if they would be willing to host a robot kitten in their homes for the duration of the study. Participants were given two different dates on which they could pick up the robot kitten at the university. After week 6 of the study, we emailed participants to schedule an exit interview. The first author conducted the interviews using a semi-structured interview protocol. The semi-structured interviews lasted about 40 min on average and included prompts about interactions and experiences with the robot kitten throughout the 6 weeks. We asked participants to share their thoughts, retrospectively, about the robot kitten from their preconceptions to how their actual interactions may have changed throughout the study (e.g., “What did you think this study would be like?”, “Tell me about the unboxing of the kitten.”, “Did you show the robot kitten to anyone?”, “How often did you interact with the kitten?”, “Did the robot kitten help with stress or provide comfort?”, “Tell me about your positive/negative experiences with the robot kitten.”). In general, the interview questions progressed from earliest to most recent and ongoing experiences with the robot kitten. Each interview was video recorded, transcribed, and de-identified, which resulted in 156 pages of single-spaced text. After the interview, we thanked participants and asked if we could contact them in 6 months.

### 3.4 Data Analysis

To analyze the rich qualitative interview data, we utilized a phronetic iterative approach that focused on the participants’ words but at the same time recognized that the research team was sensitized by concepts such as novelty and well-being (Tracy, 2020). Although “researchers invariably arrive at data analysis with some sensitizing concepts and research questions that serve as a lens throughout the process” (Tracy, 2020, p. 219), Tracy suggests that the initial analysis occur apart from those sensitizing concepts. In other words, we framed our study around issues of novelty and well-being, but our approach to analysis prioritized the data and not a predetermined lens. Once the experiences of our participants began to coalesce around specific categories we were able to view those results, specifically, as they related to novelty and well-being. This approach allowed us to give primacy to the data and participants’ experiences, in their own words, while also reflecting on previous scholarship and adding to the ongoing narrative in the discipline. This approach can create “use-inspired, practical research that not only builds theory, but also provides guidance on social practice and action” (Tracy, 2020, p. 210). To that end, we used two cycles of coding (primary-cycle coding and secondary-cycle coding) to engage the data set.

We followed a step-wise, iterative approach to analysis (Abendschein et al., 2021a). During the first step, each author read the nine interviews and then wrote a memo reflecting on the data set as a whole as well as ideas about emergent themes. Next, the team met to discuss initial thoughts guided by our general research question (i.e., “What were people’s experiences with the kitten robot?”). During these conversations, themes began to emerge. As a result, we each took a another pass through the data that was informed by our focused research question (i.e., “How, if at all, did the robot kitten influence people’s experience with novelty and well-being over 6 weeks?”).

Next, the entire team engaged in primary-cycle coding, where we assigned short phrases that captured the meaning or action of each section of the interview. Around 15% of the data set was placed in a unique file for this step. Each author was responsible for coding the entire file and generating initial codes based on the action in each line or segment of text (e.g., comforting, friends/family, excitement, newness, etc.). After this process, we met to discuss and reflect on the initial codes. Using these insights, we developed a codebook with six categories with definitions and examples for each. We applied this codebook to the entire data set, with each author coding three interviews. We created a unique file for each of the six codes and put corresponding quotes from the interviews into those files (also known as axial files). We met regularly to discuss any questions/issues with our progress.

Next, we engaged in secondary-cycle coding to examine the potential for connections between the categories as well as relevant literature. We each read through the axial files and then met to collectively interpret the data. The goal of this step was to “construct explanations for the participants’ explanations” (Tracy, 2020, p. 7). We continued to keep the data central to our analysis and met regularly to discuss the categories and subcategories that we saw emerging across our analysis. We utilized analytical memos during this entire analysis to reflect on the participants’ words, foster discussion, and build arguments. The ongoing discussion among team members about the data and the sharing of memos helped our “awareness about [our] actions, feelings and perceptions” (Darawsheh, 2014, p. 561) related to these interviews. Any disagreements about the data were resolved through discussion and reflection. Axial files, interview questions, and a sample author memo are available on OSF. Please see Data Availability Statement at the end of the manuscript.

## 4 Findings

**FIGURE 2:**
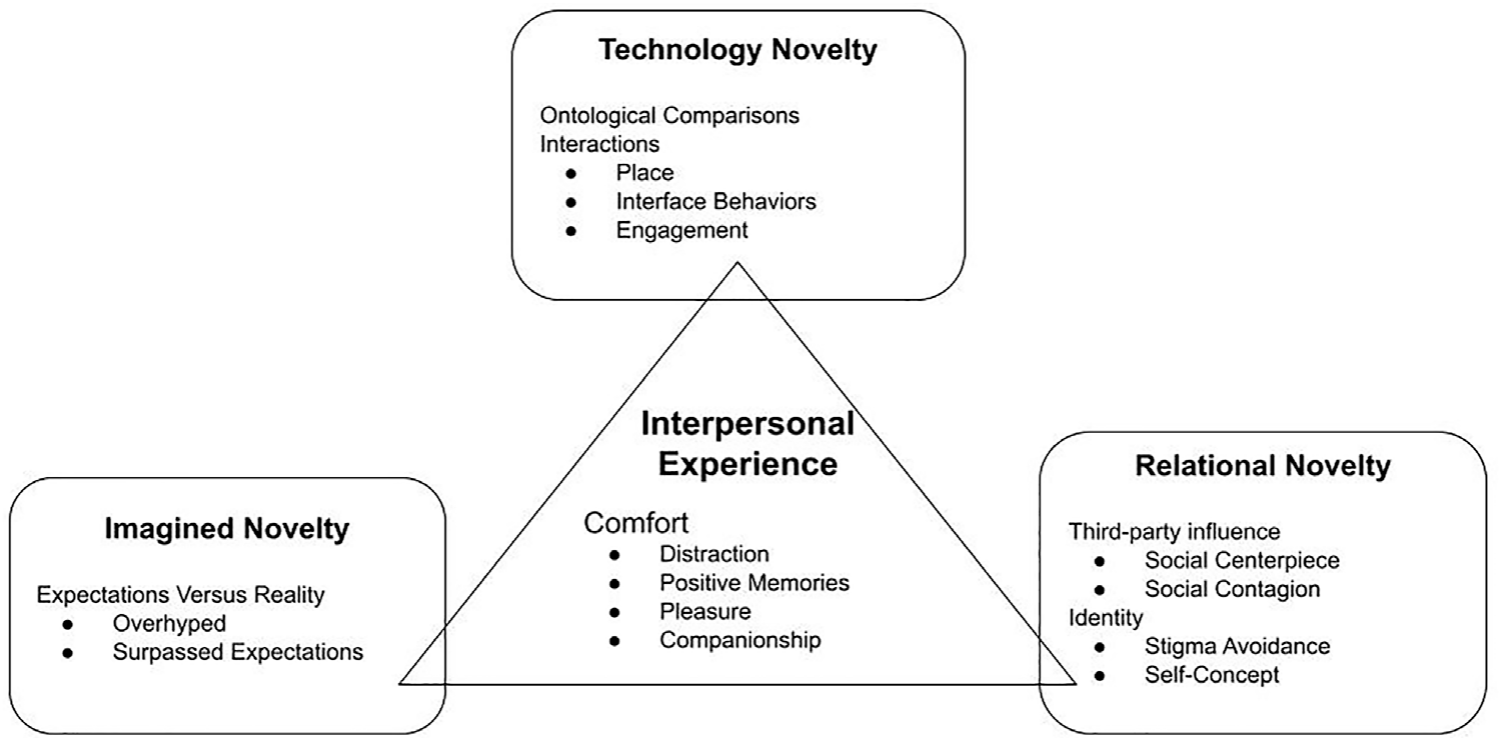
Novelty Types. Technology novelty refers to participant interactions with the robot kitten where they tried to figure out its purpose and functions. Imagined novelty emerged when participants measured their expectations versus reality when meeting the robot kitten for the first time. Relational novelty is represented in our data through interactions with others about the robot and reflexive interactions that were influenced by an actual or perceived social judgment from others. Our findings also suggest that these three elements of novelty are related to positive interpersonal outcomes.

In this study, we sought to understand novelty-relevant experiences surrounding in-home companion robot pets for adults that were living in some degree of social isolation due to the COVID-19 pandemic. We suggest a new conceptualization of novelty as an ongoing process involving imagined novelty, technology novelty, and relational novelty, with each component related to the interpersonal experiences of the user (Figure 2). Specifically, we used an iterative approach to data analysis with our focused research question, “How, if at all, did the robot kitten influence people’s experience with novelty and well-being over 6 weeks,” that helped us identify the four categories in our model of novelty as an ongoing process with six emergent themes related to how our participants experienced the robot kitten (Figure 2). The data suggest that imagined novelty involved expectations that did not always match reality, whereas technology novelty included participants comparing the robot kitten to other entities. Relational novelty, on the other hand, covered the third-party interactions that influenced the way participants perceived the robot kitten, but also included self-disclosed aspects of identity that emerged when discussing their experience. Each of these seemed to be related to the interpersonal experiences of our participants specifically related to how this robot was or could be comforting. We elaborate on each of these categories and themes in the following sections by highlighting the words of our participants, suggesting connections within the data set, and linking our findings to the broader literature.

Our findings highlight the salient experiences of participants as they lived with their robot kitten for 6 weeks (Table 1). Each person talked about how this new experience of adopting a robot kitten contributed to unique interactions with friends, family, and other animals. Although the findings are specific to our sample, it is quite likely that the results could be transferable to other adults living in social isolation. As a note, quotes have been lightly edited for length and readability (raw, unaltered quotes can be found on OSF, linked above). Edits, however, did not impact the meaning conveyed in each quote.

**TABLE 1:** Number of participants per theme.

Category	Number of participants represented
Expectations versus Reality	9
Overhyped	5
Surpassed Expectations	5
Ontological Comparisons	9
Interactions	9
Place	9
Interface Behaviors	6
Engagement	8
Third-party Influence	9
Social Centerpiece	6
Social Contagion	5
Identity	8
Self-concept	5
Stigma Avoidance	7
Comfort	8
Distraction	4
Positive Memories	3
Pleasure	5
Companionship	4

Sum total of subcategories may exceed the number of total participants since the subcategories were not mutually exclusive.

### 4.1 Imagined Novelty

We define *imagined novelty* as the assumptions or preconceived notions that exist prior to interacting with a machine or new technology for the first time. Overall, our participants began the study with excitement and uncertainty about the robot kittens, in line with people’s responses to novel technologies, more broadly (Smedegaard, 2019). During the exit interview, we asked individuals to reflect on what they thought living with the robot kitten would be like and how those preconceived notions aligned with their actual experience. Across interviews, we heard participants talk about what they imagined the robot kitten would be able to do, how it would sound or look, and even how they would feel when they met for the first time. Based on those experiences, we use the term, *imagined novelty*, to describe what emerged when participants discussed their expectations versus reality when meeting the robot kitten for the first time.

#### 4.1.1 Expectations Versus Reality

Participants brought various expectations about how the robot kitten would look and certain functions that it might be able to perform. These expectations, however, did not always align with reality as people met their new companions during the unboxing. Although a few people noted that the robot kitten was close to what they predicted (e.g., “I’d say it was pretty on par with what I would have expected from it” and “when I pictured a robot cat, [it’s] kind of what I pictured in my head”), the majority commented that the robot kitten was quite different from what they had imagined. The data suggested that participants were likely to face either disappointment or pleasant surprise when they first encountered the robot kitten, tendencies we have termed overhyped versus surpassed expectations, respectively.

##### 4.1.1.1 Overhyped

Before receiving the robot kittens, many participants had high expectations for their physical appearance and behavioral capabilities, often referencing media portrayals of sophisticated robots that evoke wonder (e.g., Boston Dynamics). However, the reality of unboxing the robot kitten often failed to live up to the “hype.” Several participants overestimated the abilities and appearance of the robot kitten prior to their first encounter. Lofty expectations often generated a sense of feeling underwhelmed (e.g., “I just wish it did more things… like, if it could like walk or something. That’d be kind of cool. Or it if could initiate interaction,” “I guess I had a little bit higher expectations,” “As much as it looked realistic and could do some realistic things it just felt very fake and artificial,” “[I thought it might be] partly alive, kind of, like that was my mindset going into it and so, I kind of, guess I, kind of overhyped [it] in that sense”). Individuals thought it would be a new experience that brought with it a sense of excitement; instead, it let them down a bit.

Initial expectations of the robot kitten appeared to be informed by past experiences with other robots and/or real animals. Participants brought an idea of what the robot kitten should look like and how it should be able to function (e.g., “I was picturing, like, one of those sleek, you know, like, um, no, fur on it, but like the white little robot, like walking around meowing and just kind of being like, those little flip cats”). The expectations were even higher for those who had a real cat in their life. For example, one participant explained:

I guess coming from where I am, back at my house, I have three cats myself. So, I kind of expected it to do a little more cat-like things. I don’t know, I just kind of like, the way I hyped it up to be from having three cats, just like, [did] not meet that expectation (Interview 7).

This participant had lofty expectations for the robot kitten and seemed to overhype its potential based on past experiences with a living animal. Meeting the robot kitten was underwhelming and did not generate a sense of newness and excitement, since it failed to achieve the expectations set ahead of the unboxing. The impact of the participants setting lofty expectations and then confronting the reality of a kitten robot that could not walk or initiate interaction was a sense of disappointment and diminished enthusiasm, even when they acknowledged liking the kitten robot.

##### 4.1.1.2 Surpassed Expectations

Not everyone was disappointed about the way the robot kitten looked or its limited functionality, as exemplified by the participants, including the one who experienced it as “surprising, but in a good way.” In contrast to overhyping the form and function of the robot kitten, several participants noted that the initial encounter surpassed their expectations. They seemed excited by the difference and enjoyed that the experience was not at all like they had envisioned. One participant commented, “I expect[ed] it to be a little bit different. So, I was surprised when it was such a cute little kitten. When it was all furry and, like, purring and I was like, oh, okay. This is different.” When thinking back on the experience of meeting the robot kitten for the first time, another participant recalled their expectations and the surprise when they encountered something different:

I don’t think [my roommate or I] either of us expected, for some reason, I guess we didn’t think it was going to have fur. I don’t know why I kind of thought it was going to be like, white, you know, plastic-type stuff, but, I was I was pretty surprised in a good way though it was pretty cute (Interview 9).

An interaction that exceeded expectations in a positive way (e.g., “it responded quicker than I thought it would” and “I mean, my first impressions it was definitely was like, oh, it’s really soft”) was enjoyable and fed peoples’ interest. For example, one participant said, “It definitely made me want to get more social robots cause it makes me want to, like, find out more about them because I just find it really fascinating.” In this way, the robot kitten seemed to initiate a sense of wonder not previously experienced or anticipated.

##### 4.1.1.3 Summary

All participants spoke to the relationship between expectations and reality when initially experiencing the robot kitten (Figure 2); typically in terms of a gap or misalignment between expectations and reality that influenced their reactions toward the robot kitten. Their expectations were influenced by media, prior experience with other robots, or the zoomorphic nature of the kitten robot (i.e., a form-function attribution bias, (Haring et al., 2018). Both the experiences of 1) overhyped novelty followed by disappointment and 2) assumed familiarity followed by pleasant surprise demonstrated that novelty is valued in initial HRI and that users bring novelty expectations that may be positively or negatively violated. This imagined novelty can lead to expectancy violations which may lead to positive or negative outcomes, depending on people’s appraisals of their meaning (Burgoon, 1993). Previous research shows that interaction with social robots may be influenced by expectations (Kwan and Fiske, 2008; Spence et al., 2014; Edwards et al., 2016; Edwards et al., 2019; Horstmann and Krämer, 2019) and these results suggest the importance of examining how expectancies and expectancy violations may shape imagined novelty in HRI.

### 4.2 Technology Novelty

We define the term, *technology novelty*, as the interactive, sensemaking process where people work to determine the purpose and functions of a machine or new technology. Our participants seemed to engage in this ongoing endeavor of trying to understand the abilities and potential impact of the robot kitten, particularly when it came to their unique life circumstances. The data suggest that individuals thought about how the robot kitten was similar or different from other technologies in their lives and that those thoughts influenced how they viewed the robot kitten. Their conceptualizations of the robot kitten also seemed to shape how they interacted with the machine throughout the study.

#### 4.2.1 Ontological Comparisons

Across interviews, participants invoked comparisons to other more familiar categories of being when thinking about their novel experiences with this new technology. For example, they related the robot kitten to “real” cats or other nonhuman animals (e.g., “That’s like my cat back home”), technologies like the mobile phone (“[it’s] like, getting a new phone. It’s just a new phone to me), commercial or literary robots (e.g., “Like, the Boston dynamics one, yeah… those things are very responsive” and “I’ve seen ones from, like, Sony where they can walk around”), and toys (e.g., “I feel like [if] they responded back a little bit it would have more life to it like Fubies” and “like a the FurReal Friend like, it’s very similar”). Participants articulated the similarities and differences between the robot kitten and these other known entities, often drawing upward comparisons that stressed what the robot kitten lacked in relation to other, more “advanced” entities. For example, in contrasting the robot kitten with Marvel’s Vision character, one participant explained: “the kitten robot, it doesn’t do too many actions. But then you look at Vision, and he walks around, he talks, like, he has his own thought process, which, he’s a computer, but it’s a thought process. It’s, he’s, a lot more. I think it’s a lot more complex.” When confronted with novel partners, as is often the case in HRI, people may seek to draw boundaries around and between these new forms and older, more recognized entities in a process of ontologizing, or attempting to understand the fundamental nature of beings (Severson and Carlson, 2010; Kahn et al., 2011; Edwards, 2018).

Participants also used comparisons to better understand where the robot kitten fit into their lives. In the process of prototyping in HRI, people try to answer the question “what is it?” by attempting to classify an unfamiliar actor or artifact within an overarching category, which then aids sense-making and guides interpretation and behavior towards it (Edwards et al., 2019). Some participants found comfort in situating the robot kitten in relation to familiar memories or objects. These types of comparisons also revealed a struggle to find a balance between what was familiar and this new experience with the robot kitten. One participant articulated this point by saying:

Maybe just because it only does do a few things that it’s not like a real cat in a sense where, like and then obviously, since it’s like, in the shape of a kitten and it just kind of like, maybe triggered like those memories of, like when my real cat would come and lay with me and I’d be able to pet it. And so maybe for a time I was able to like imagine that “okay, this is a real cat, and I can pet it and It makes me feel good” (Interview 8).

Others echoed this style of sensemaking by comparing their interactions with the robot kitten to other interactions with animals. holding the robot up to other living things, interactional norms, or personal memories (e.g., “Winning over its love because cats are super stubborn and so it just feels like kind of boring to just like have a cat that already just like, likes you or loves you in a way” and “I don’t know, basically, just excited to try it out and see what it was like and see if it was like, just as good as the real thing or not”). The process of sensemaking seemed to include outwardly looking for things to compare the robot kitten to, as well as reflecting on past experiences.

##### 4.2.1.1 Summary

By comparing the robot kitten with other familiar entities, our participants demonstrated that initially, in the early stages of encounter, sense-making, and experience, novelty can be generated in the space between known and unknown, where the robot kitten was understood as distinct enough from other entities to be seen as new but similar enough to make sense. Participants expressed wanting the robot kitten to be more like certain types of being (e.g., a cat) and less like others (e.g., a toy). Their attempts to understand the new robot kitten required relating it to the familiar and finding points of convergence and divergence. However, they described a lack of novelty when the robot kitten was perceived as overly similar to something they already knew (“I mean, that’s the kind of thing you see with a Furreal Friend, it’s very similar…it’s like oh, okay”). In this way, our results suggest that the experience of novelty emerges from simultaneous confrontation with known and unknown, sameness and otherness. Perhaps novelty exists in the optimal space or “Goldilocks zone” between known and unknown in which a robot is perceived as not too similar (uninteresting), and not too different (unintelligible).

#### 4.2.2 Interactions

Despite overhyping the robot kittens initially, or having their expectations violated, participants still reported interesting and enjoyable interactions with this new technology. This category focused on the affect, behavior, and cognition related to direct interaction with the robot kitten. This included thoughts and feelings about interactions as well as bonding aspects that might have led to a change in engagement or interpersonal evaluation of the experience or this type of technology (i.e., robot companion pets) across subsequent interactions. For example, participants reported interacting with the robot kitten regularly at first and then saw those interactions taper off. As one participant noted:

I think at the beginning, it was pretty exciting. It was something new. I mean, we’re all adults, but it was like a new toy to play with for a while. So that was exciting. But, definitely after a while, it’s not that maybe the robot got boring, but, we, it just kind of blended into the rest of my life again. It wasn’t new and shiny (Interview 1).

Here, the participant notes that the interactions were enjoyable, but over time those interactions and the robot itself became integrated into the daily life world, becoming less salient and exciting. This participants’ description of experience echoes central aspects of technology domestication in the sense that the new technology was first marvelous and strange (Baym, 2015) and then tamed through integration into daily practice, with ensuing adaptation and meaning-making processes (Silverstone and Hirsch, 1992). In the process of technology domestication “artefacts need to be acquired (i.e., bought or in some way made accessible), placed (i.e., it is put in a mental and/or physical space), interpreted (i.e., to be given a meaning as well as a symbolic value to the outside world) and integrated into social practice of actions” (Sørensen et al., 2000, p. 240). Although every participant interacted with the robot kitten, and those encounters varied across interviews, participants specifically talked about the place where the robot lived, the different behaviors they experienced, and how they engaged with the robot kitten throughout the study.

##### 4.2.2.1 Place

Placement of the robot within their homes took on a special significance, as participants described where their robot kitten “lived.” The data seem to suggest that as novelty related to this new technology evolved, participants also reconsidered where to keep the robot kitten. These changes encouraged different types of interactions as the robot kitten was often moved from shared spaces (e.g., living rooms and kitchens) to those that were more private (e.g., bedrooms). Participants often referred to the place where the robot kitten lived when they thought about the nature of their interactions (e.g., “It primarily just lived in our living room… sometimes it was on the couch, and sometimes it was on, um, like a coffee table it, depending on who was holding it last I suppose where it ended up sitting” and “It lives on my couch for the most part”). Participants were also aware of its place and recalled thinking of the robot kitten even when it was not around (e.g., “I feel bad because right now it’s just sitting in my car”). One participant said:

So the first few days I feel like I interacted a lot. I mean, even to the point, I was like, I was sleeping and I could be like, oh, it’s just out in the living room by itself, I should bring it in here (Interview 8).

The robot kittens were integrated into the everyday activities associated with the various spaces in which it lived, including watching television, hanging out with family or friends, playing with pets, typing at a computer, sleeping, and meditating. Often, the robot kitten first lived in common and social spaces like the living room or kitchen and was later moved to the more personal or private space of a participant’s bedroom or desk. For some, placing the robot kitten in a common area allowed people to interact with it regularly. When it lived instead in their room or other personal space, interactions were more private and personal (e.g., “It kind of sat on my desk with me … every once in a while, I was like getting tired of working on my work with school I would go over and I would pet a cat”). One participant articulated the change in placement from a public location to a private one:

So after that, it went with me up to my room and it’s now living next to my bed on top of my mini-fridge. So, I kind of like, and the way I look at it, it’s not necessarily sleeping in my bed, but it’s more so, like, guarding my bed. Yeah, for the first week, week and a half, I interacted with it quite frequently. Also, just like having internal conversations with it in per se. So, I’d be like, kind of just like talking. Not really per say to myself, but have like a conversation with the cat. About just like, should I do this? Should I do that? Or I was just like, what I should eat, of just like, normal conversation I would have (Interview 7).

Moving the robot kitten from room to room or to different locations throughout the study was common. However, the affordances and interactions changed when the robot kitten moved from the common areas to ones that carried more restrictive access.

##### 4.2.2.2 Interface Behaviors

Even though the robot kitten had limited movements and vocalizations, participants regularly commented on its interface behaviors and design features as central to their experiences. In describing their interactions with the robot kittens, participants emphasized the enjoyable haptic interface of touching soft fur, holding it in their lap, and feeling it purr and knead. The robot kitten’s behavior encouraged positive responses as well as thoughtfulness about their emotional reactions to specific actions (e.g., “I like when it purrs, I feel like that’s the best part of it because I think it’s cute,” and “It was just kind of fun to have a meowing kitten robot on your lap.”). One participant captured both of these elements, explaining:

I would always pet it and try and [try to] figure out how to make it purr though because I don’t love, I don’t love the meow and, but I like purring, the purring is cute. I would have it on, but I would try and like, I don’t know, manipulate it. Make it only purr. I don’t know. I couldn’t really do it that well (Interview 8).

The interface behaviors seemed to enhance interactions with the kitten robot, at least initially (e.g., “I think that if the robot kitten had been a little bit more advanced and had, like, more features. I think I would have interacted with it more and it would have gotten my attention a little bit more”). For some, when the behaviors became mundane or predictable, they still held out hope that new actions or experiences would emerge:

I kind of was holding on to this idea that it would gain new, like, actions. Or, like, it would be some kind of learning machine, but I have not experienced it having new actions. And so, kind of after a while, when you have like this, when you get a new technology, no matter what it is, it’s going to be super exciting and you’re going to be like, yeah, I have this thing, but then, you know, after a couple of days, I guess it kind of loses its shine and sparkle specifically when you figure out all of its functions and it doesn’t do anything else. And I guess in a way, the cat didn’t have like, a specific function for me that made it more worthwhile to use, like, to actually use every day. Especially when it didn’t do anything new and I couldn’t discover anything more about it (Interview 3).

The potential for new behaviors to emerge kept this participant hanging on in hopes of experiencing a greater level of interaction.

##### 4.2.2.3 Engagement

Early interactions with the robot kittens were characterized by intense observation (passive engagement) and experimentation (active engagement). Participants tried to quickly learn all of the different things the robot kitten could do, like “at the very beginning, when I was trying to figure everything out [and] pretty much left it on the whole time.” Other participants echoed the process of rapidly building understanding of the robot kitten’s behaviors through involvement and exploration, for instance: “When I first got the kitten I, uh. I feel like I very quickly learned all of the things that it could do,” and “So, we kind of like, looked it up and figured out what it was and stuff and we were like, okay, I, I see what this is about now.” This distinction between passive and active engagement emerged throughout the data set. Participants talked about their intentional interactions with the kitten robot (e.g., “I consciously think about it so I tried to [interact with] it pretty regularly throughout the 6 weeks” and “I would pet it and then it would start purring. … it was nice to use it as, like, a way to get a break from school work) as well as those that were less intentional (e.g., “you know, you might bump it. And then it meows, and you’re like, oh, right hello kitten”). Participants thought about the robot kitten initially and then it seemed to fade into their lives, but they often continued their engagement even if it was unintentional because the robot was a part of their world.

These processes entailed simultaneous confrontation with novelty and predictability; paradoxically, the quest to discover novel responses led to the experience of their diminishment. For instance, one participant explained: “I don’t know. If the actions were kind of more random [the robot kitten would have held my interest longer] because everytime that you’d would interact, it would take, like, be a full 4 min for it to fully go through his entire cycle of, like, meowing, kneading, meowing, and then purring. Just, like, kind of like, stuck to that, like, lock lineage of actions in a row with nothing varied.” As participants progressively charted the limits of designed and programmed capacity to the point they were no longer surprised by the robot’s behavior, the robot “lost its shine,” or its “initial like shock value,” to quote two participants, and became just another thing in the room.

At the same time, the seemingly opposite was true: The pursuit of predictability, or attempts to “figure it out,” could generate novel experiences as participants confronted new and unknown features, behaviors, and patterns of interaction in the process of becoming familiar with the robot kitten and integrating it into their home lives. As recalled by one participant: “Well, I think 1 of the first nights we were watching TV, my mom and I were, and my sister came in and asked about the cat. Because it was just sitting on the table and then we were like, oh, right the cat. And so we picked it up and I was holding it in my lap, on my lap. And that’s when we kind of realized that there were different reactions when we were petting it, like, it’s not just the same meow every time there’s different meow patterns, and we ended up pausing the movie because, uh, we wanted to see what different meows would happen.”

Despite having a limited mechanical ability, participants regularly talked about their engagement with the robot kitten as ongoing (e.g., “Sometimes I’ll accidentally brush up against it and it will meow and I’m like, oh, hey”). Nearly everyone seemed to start out intrigued by the robot kitten and took the time to figure out the functions (e.g., “I think the most surprising thing was when we turned the, uh, thing on, and it started moving its paws a little bit. That was pretty funny”). Over time, however, participants reported the newness faded and with it their interest. One participated recalled this transition when they said:

I don’t know if it was sort of a novelty thing, you know, it was new and fun for a little bit and then it sort of lost its, you know, I don’t know the right word for it, like, interest or whatever, it was kind of cool enough to where it didn’t completely dissipate, but, you know, it’s, at a certain point, we kind of had other stuff to do and talk about … I think it’s the same with the interest in the sort of maybe, you know, it was on a downward slope, but I guess it probably sort of leveled off. (Interview 9)

The declining engagement seemed to stem from fading interest from participants, but also from the busyness of life. Overall, participants seemed to think about the kitten robot in the midst of their hectic lives even as the newness faded (e.g., “I guess I still have not really given up the hope that it might learn a new thing or do something different, and I’ll be like, oh, my god, it did something new for me”).

##### 4.2.2.4 Summary

Our participants’ interactions with the robot kitten-in terms of placement, interface behaviors, and engagement-highlight the dialectical, or tension-filled interplay between novelty and predictability/familiarity, as well as the dynamic and nonlinear aspect of novelty experience. From a dialectical perspective, novelty and familiarity can be experienced only in relation to one another, forming a unity-in-opposition (Baxter and Montgomery, 1996). Although novelty and familiarity are contradictory, individuals may desire them simultaneously from relationship partners (Baxter and Simon, 1993; Baxter and Montgomery, 1996) and societal discourses promote both novelty and familiarity as legitimate individual needs and features of good relationships (Baxter, 2010).

Overall, the process of sensemaking that our participants experienced seemed to contribute to the larger idea of technology novelty (Figure 2). Technology novelty relates to the intentional interaction with the robot kitten as participants sought to figure out what it was and see how it worked. In contrast to the way novelty is often presented in the HRI literature (i.e., technology novelty is the only type of novelty), we argue that there is more to the novelty story.

### 4.3 Relational Novelty

We define *relational novelty* as the experiential impact of an actual or perceived social judgment from others on one’s view of a new machine or new technology. Participants talked about how individual and shared interactions with the robot kitten influenced their perspective of the experience and their own self-concept. Introducing the robot kitten to others gave participants an opportunity to gain additional perspectives that oscillated between encouraging/intrigued (e.g., “[My parents] honestly thought it was kind of funny and cute” and “I had told [my roommates] about it and they thought it sounded kind of weird, but they were also kind of intrigued to see what it was about”) and discouraging (e.g., “I introduced it to my parents, my brother, and a couple of friends… I was so excited and then everyone was just like, yeah, well that’s not that amazing.”). Regardless of the outcome, these encounters allowed our participants to share the experience with others and see how their friends and family responded to the robot kitten. Likewise, aspects of living with the robot kitten for 6 weeks led some to think about their own life circumstances and how adopting a robot companion pet may be better suited for someone else (e.g., “I would say I’m doing pretty fine in my life… But, maybe [for someone who] doesn’t get as much social interaction… I think it could help them a lot more and maybe they’d be a lot more into it”). Taken together, the influence of others on the experience and the reflection on one’s own identity and life situation led us to suggest that *relational novelty*, represented in our data through interactions with others about the robot and reflexive self awareness, was influenced by an actual or perceived social judgment from others. In other words, the robot kitten acted as a catalyst for interaction and consideration of who the self was in relation to others throughout the experience.

#### 4.3.1 Third-Party Influence

In addition to the importance of interactions with the robot kitten, encounters with others (e.g., friends, family, strangers) shaped impressions of this experience for our participants. Individuals regularly reported interacting with others about the robot kitten and how those different conversations influenced their views of the overall experience. They noticed that the robot kitten was often a conversation starter and that their interactions with others shifted the way they viewed their new pet across the 6 weeks.

##### 4.3.1.1 Social Centerpiece

The robot kitten was a catalyst for new conversations which introduced novel social interactions. Participants often introduced the robot kitten to others in their life and found that it served as a social centerpiece. In other words, it got people talking, laughing, and thinking about social robots in general (e.g., “Whoever dropped by our apartment. They would like to see it and be like, what is that? And so then I’d have to go through the whole explaining it, and it was still interesting to just explain it to people). These interactions were often positive even if people thought the premise of a robot kitten was silly (e.g., “I had a picture on my phone [of the robot kitten], and I also had a video of the cat kneading. And so I passed that around. And my grandpa thought [the robot kitten] was kind of weird. But my dad and his fiancé thought it was neat”). Participants were delighted to introduce their robot kitten to others, as one participant commented:

So, whenever we had guests over, we would say these are our cats type of thing. I named my cat Meatball, so I’d always introduce people to Meatball. I think the first person we had over was a mutual friend of ours and he laughed at the name and he wanted to know more about the study and, you know, what it was meant for that sort of thing, he seem[ed] pretty interested. He picked it up, kind of a, you know, [they were] laughing and, you know, shock kind of, when it started meowing, I think, was icing on the cake. And it was kind of funny for them… I was sort of taking the robot at face value and well, it did have some stress relating factors, I guess. I didn’t consider the value it brought in, you know, creating conversations and room for thought (Interview 9).

As a social centerpiece, this participant highlighted the utility of the robot kitten to reduce stress levels, as well as its ability to be a catalyst for novelty in interactions with others. Another participant recalled opening the robot kitten on a video chat with some friends: “We just kind of sat on a video chat, you know, we test[ed] out all the different features. We did that together and it was a lot of fun.” The robot kitten was not only a conversation starter but also a social touchstone that people gravitated towards during interactions.

##### 4.3.1.2 Social Contagion

Across interviews, participants noted that their interactions with others influenced their impressions of the robot kitten. In this process of social contagion, participants were highly sensitive to third party reactions. Some noted that their conversations with others made them less excited about the experience. For example, one participant talked about how their initial enthusiasm diminished as she interacted with others:

I was at my boyfriend’s house when I unboxed [the kitten robot] and I was the one who was telling [my boyfriend] all of this stuff like oh, I think it’s going to be awesome. It’s going to be like a learning robot and this and that and I unboxed it and he was like, “[the robot kitten] has a hairbrush.” I was like, I know it has a hairbrush, but I, it’ll be good. It’ll be fine. And then I turned [the robot kitten] on, he was like, “is that all it does” and I was like, stop ruining it for me. So, uh, according to my boyfriend, it was very underwhelming, but I was excited still at first just kind of, my optimism goes down a little bit in each [round of the] study, which makes me feel sad because I was really excited. I think I let people get in my head too much about it (Interview 3).

In this way, when others were critical, unimpressed, or bored, participants could experience a loss of interest in the robot kitten.

Conversely, the robot kitten could become renovelized through vicarious experience when a third party interacted with it for the first time and appeared impressed and engaged, in line with studies showing the effects of other people’s messages about robots on individual experience (Liang and Lee, 2016; Edwards C. et al., 2020). Participants commented that their interactions with others injected excitement and enjoyment into their experiences. One participant remembered the drive home after she first picked up the robot kitten:

I picked it up with my mom and my sister and we didn’t wait until we got home to start unboxing. We parked in a parking lot kind of near where we picked it up, we walked over and as soon as we got to the car, we started opening the box cause we were just very excited to see it and so the ride home was spent, I was in the passenger seat, and I had the kitten robot in my lap and we were laughing at how loud it was meowing and we were petting it. And so, it was very exciting on the ride home (Interview 1).

Beyond the initial excitement, there was also a renewed interest as participants introduced the robot kitten to other people. For example, when a friend was visiting from out of town and inquired about the robot kitten, one participant recalled the story of how their friend was hesitant but, “We told [him] to pet [the robot kitten] and it just started meowing and moving its feet. He was kind of taken aback for a second, but I [think] he thought is was kind of cool.”On one hand, interactions with others seemed to stifle the excitement that participants felt about this whole experience. Whereas others reported feeling a renewed interest at various points across the 6-week study when they introduced the robot kitten to others and talked about their experience (e.g., it’s definitely a conversation piece whenever we have people over”). One participant remembered sharing the experience with her sister and niece:

My niece is six, so she was really interested and she wanted to see it really badly. So I brought it over to their house and she really enjoyed playing with it. She thought it was really cool cause her dad’s allergic to cats, so she’s never been able to have one. So she thought it was really cool and she wanted to play with it. Across interviews, participants talked about how their interest in the robot kitten was influenced by interactions with friends, family, and even strangers.

##### 4.3.1.3 Summary

From the overarching theme of third party influence emerged two critical insights about novelty experience in HRI: 1) that novelty is a renewable resource 2) and that experience may be renovelized and denovelized through social interaction or subjective experience. Participants recounted how social interactions with third parties (parents, friends, roommates, strangers, and pets) replenished or depleted the robot kitten’s novelty for them. Several participants articulated the value of the robot kitten in terms of its use as a talking point. As a “conversation piece,” the robot was used to foster novelty in interaction with other people (or with pets, when it was presented as a toy or experiment). This result was consistent with research demonstrating that HRI may facilitate and mediate human social interaction and that people may exercise interpretive flexibility in their use of zoomorphic social robots (Šabanović et al., 2013; Chang and Sabanovic, 2015). Across diffusion of innovation and technology acceptance models, there is recognition that technology adoption processes are inherently social and may be influenced by others (e.g., Straub (2009); Rogers, 2010) and altered through social interactions to affect behavioral intentions and uses (Venkatesh et al., 2003; Alaiad and Zhou, 2014). Whereas the majority of this work has centered on the importance of third party influence and subjective norms on the decision to use and specific use practices, our participants shared experiences relating the ways in which, even after a robotic technology had been adopted and used, subjective interpretations of its value, newness, and meaning were responsive to shifts based on the opinions and reactions of others.

Importantly, the effects of third parties were often described by participants in terms of their influence on levels of interest or excitement toward the robot kittens and not explicitly on the discovery of objectively novel behaviors or features. However, across interviews, participants used adjectives including “new,” “shiny,” “shock value,” “exciting,” “sparkle,” and “cool” (terms clearly characterizing the experience of novelty) in contrast with their experiential opposites of “boring,” “loss of interest,” “underwhelm,” and “dissipating” engagement. Thus, whereas novelty in their earliest encounters with the robot kitten was more tied to the nature of the technology and its behaviors as previously unknown to them, the emergent practical understanding of novelty experience after initial interaction was synonymous with interest and engagement (i.e., opposite of boring). For this reason, the significance of re- and de-novelizing third-party effects was on their ability to manifest changes in participants’ interest and engagement with the robot kittens. This mirrors the sort of novelty experience that human partners in ongoing relationships with one another may encounter. Here, novelty experience does not require discovering something previously or completely unknown about the other but may emerge when partners see each other or their relationship in a new light that is based on new shared experiences, shifted perspectives, or the influence of social networks (Baxter and Montgomery, 1996).

More broadly, the third party novelty effects described by our participants underline the link between human sociality and perceptions of technology. In the case of novelty, HRI expands beyond direct interaction between one human and one robot to encompass relational novelty (Figure 2) with human-human interactions about robots, onlooker observations of other humans interacting with robots, and human-human interactions facilitated by a robot. Relational novelty encompasses the social processes through which other people contribute to the perception of newness and levels of excitement and interest toward a social robot. Third-party influence can also impact our experiences of renovelization and denovelization. In other words, it may be that our relationships help deplete or replenish novelty over time.

#### 4.3.2 Identity

Identity is relational. In fact, relationships involve “a process of coordination that precedes the very concept of self” (Gergen, 2009, p.xv). In other words, people develop a unique view of the world and their identity through interactions with others, or in this case, a new machine. This behavior aligns with our definition of relational novelty. The data suggest that participants reflected on their potential for a relationship with the robot kitten specific to its function and purpose and how those elements mapped onto their current self-concept (i.e., how they viewed themselves). With that in mind, personal identity emerged as an important factor that participants faced when determining whether they would use and benefit from this new technology in the longer term. Participants talked about how others may perceive their use/enjoyment of the robot kitten as well as who they thought would be ideal users for this type of technology. They attempted to avoid social stigma through privacy management (for instance telling only a select group of people about the robot kitten) and framing the robot kitten as “useful for others, not someone like me.” Both of these elements included a level of self-evaluation on part of our participants that was connected to their relationship with the robot kitten.

##### 4.3.2.1 Stigma Avoidance

Participants sought to avoid stigma by guarding against the judgment of others. Although many people shared the details of their participation in this study with friends, family, and acquaintances, several others mentioned that they were nervous about sharing their experience, so they did not actively discuss the study with others (e.g., “Some of my other friends don’t know, I don’t tell them that kind of thing. Just, [they] might find it a little weird”). The stigma of playing with a “toy” was something that other participants mentioned being self-conscious of when interacting with others:

It’s the beginning [of the study], we decided to bring the kitten in the car with us, my mom and I, when we went and picked up food. I didn’t realize that my neighbors were getting home at the same time as us. So, my mom had a bag of food, and I had the robot kitten and I had been petting it in the car, so it was meowing and I felt a little embarrassed when our neighbors saw. Because I, I’m not sure why, maybe because they didn’t know the situation and it kind of just looked like I was playing with a toy cat. Um, so not super negative, but it was a little embarrassing at the moment because I didn’t realize that they would see me with the cat. I guess I don’t really know my neighbors very well, but they’re only a couple years older than me. And so maybe because they’re like my peers, I felt like they might think I looked maybe childish for playing with a like, a toy in front of them. It would have been different if it hadn’t of meowed, I think, but, the cat meowed and it was like, okay, who has a cat right now and the answer is no one it was a robot. Yeah, I did, I wanted to show them that it was a robot that. It wasn’t just a toy. It’s actually really cool (Interview 1).

The scenario here demonstrates that while participants accepted the fact they were in a study and may have been able to compartmentalize “playing with a toy cat,” others, outside the study, did not know the whole story. This participant’s desire to show the neighbors the kitten and explain “it’s actually really cool” was an effort to avoid stigma by clarifying their actions.

Participants also worked to avoid stigma by suggesting that the ideal demographic for this machine was not them. Specifically, they thought the robot kitten would be best for children (e.g., “I think a kid would really enjoy cuddling with it. If they couldn’t have a cat on their own. Um, but for a college student, I kind of felt like it didn’t have a purpose with me”), an older adult (e.g., “People in old folks homes it would definitely have that sense of having the animal around, but not actually having a real animal because some, it’s just not possible in some situations,” and “Maybe an older person who maybe doesn’t quite have the mental capacity that they used to have would enjoying, maybe they, I feel like maybe they would find a lot of comfort in it”), or someone with a specific illness (e.g., “I think people with Alzheimer’s definitely would [get] appreciation out of it” and “I guess, when you have dementia, you don’t really know better. You think it is an actual cat”). Participants seemed to divert the stigma of having a little robot kitten by pointing to others who they thought would be more suitable hosts.

##### 4.3.2.2 Self-Concept

When participants talked about others as ideal users for the robot kitten, they also revealed aspects of their own self-concept. Participants differentiated themselves from others by stressing their own individual or social resources. For example, by saying that the robot was best suited for those who were older, lonely, or didn’t know any better, participants implied that they were in the prime of their lives and not lonely (e.g., “For a college student, I kind of felt like it didn’t have a purpose with me”). One participant said it this way:

When you’re in college, and you’re like middle-aged and you’re figuring out life, I guess we kind, I’m trying to phrase this correctly, like, for me, it didn’t have a purpose like I didn’t need, like, I don’t need an animal because I have so much going on, but when you’re a kid or when you’re older, it’s kind of like, life is a bit at a standstill. I think when you’re a kid [or older] adult, it serves a certain purpose and then this point in my life personally, it doesn’t. People a little bit older, like the purpose it serves is as a companion because they don’t really know better at that point, you know? Kids look at the simplest things and think it’s the most amazing thing ever, “I can’t believe this.” Where I feel like as adults, like, you know, 20-year-olds [and] on, we kind of lose that sparkle where the simple things just don’t hold that much value to us anymore (Interview 3).

The identity of our participants seemed to be tied to their perceived use or purpose for the robot kitten. Many of them stated that they did not need it in their life because they had friends, social activities, and/or real animals. Implicit in participants’ explanations of the robot kitten’s ideal user were assumptions that it was either a sophisticated toy or a replacement for social contact with living beings, despite articulating the value of the robot kitten as a complement (facilitator, catalyst) to social interaction with other people and pets when describing their use practices. This phenomenon has been observed in other studies as well. For example, older adults with mild cognitive impairment indicated that assistive robots would be useful to older or more disabled people, but not for themselves (Wu et al., 2016) and older adults with mild-to-moderate Alzheimer’s disease deemed an assistive robot “useful for others whose health was worse off than their own or who were socially isolated, suggesting that they perceived themselves to be more able or better cared for than the expected users” (Wang et al., 2017b).

##### 4.3.2.3 Summary

These results concerning the identity-related issues surrounding the use of the robot companion kitten further demonstrated that assistive robots may be associated with often-marginalized identities and groups. Even though participants found the kitten robots to be “neat” or “cool” they viewed their experiences in conjunction with their self-concept, stage in life, or group identity. For example, participants associated the robot kitten, or a similar type device, with disability and aging (e.g., “I think that it definitely would fill a niche, of like, old people [that] are not as used to having technology and also people with Alzheimer’s”) and that for long-term acceptance and use people must be able to fit the robotic technologies into their life scripts and avoid stigma (e.g., “in between my age range, I don’t think it has as much utility, But, yeah, I can see [it] filling a niche of like loneliness for older people, younger people”). This also suggests that one’s vision of their personal identity is influenced by interpersonal relationships at various times in their life, or relational novelty (Figure 2).

### 4.4 Interpersonal Experience

Although the experiences of our participants differed, they all talked about how the robot kitten was capable of influencing people’s moods, social situations, and interpersonal interactions. Specifically, our findings suggest that the three elements of novelty we have proposed (i.e., imagined novelty, technology novelty, and relational novelty) are related to how people make sense of specific *interpersonal experiences*. These relational encounters and individual outcomes were often focused on the potential of the robot kitten to offer comfort to people, even if the participants themselves did not think they were the target audience. Overall, our participants seemed to take advantage of the opportunities the robot kitten offered as they interacted with others and reflected on the uses and purpose of a robot companion.

#### 4.4.1 Comfort

Despite the apparent stigma, across interviews, participants recalled experiences with the robot kitten that brought them comfort. For some, interacting with the robot kitten was a welcome distraction from life pressures (e.g., COVID-19, final exams, relationship changes), suggesting that novelty was a resource for escaping familiar stressors (Edwards A. et al., 2020), while others seemed to draw comfort from their interactions throughout the duration of the study (e.g., “It was a comfort to have the kitten with me, honestly. He helped me kind of relieve all the stress out of my system”). Participants reported that they also found interacting with the robot kitten to be pleasurable and even experienced a sense of companionship during a very intense time. Variously, these experiences more aligned with the familiarity and dependability end of the novelty dialectic involved how the robot kitten evoked positive memories of human-animal contact, brought physical pleasure and provided a sense of companionship. One participant captured this multifaceted experience when they said:

I got COVID during the middle of the 6 weeks, maybe towards the end, I don’t know. And so, my husband and I were like, quarantined from each other, because we don’t want him to get COVID. And so, I was in our room just by myself for 10 days. And I was like, that’s when I thought of the kitten again, and I was like, oh, I should bring the kitten in here with me and now I can like sleep with it. And so, I think it was helpful, maybe for, like, a couple days to, like, [to] have the cat. I was feeling like, lonely. To just like, pet it and have it like meow or purr or whatever. Have it remind me of my cat back home at my parent’s house. And, yeah, so I think it did, to a degree, help with comforting me when I was just by myself with COVID. So, yeah, I don’t think it lasted the full 10 days, but the first couple days where I was, like, extra sad that I was by myself it was good (Interview 8).

This participant noted that after setting the robot aside after a few weeks, interacting with the kitten again in their time of need was comforting. Comfort was a common theme throughout interviews, but those experiences looked different depending on the needs of the participant, as well as their individual circumstances.

##### 4.4.1.1 Distraction

The kitten robot had the ability to introduce a helpful distraction into the lives of our participants. The current study ran for 6 weeks and encompassed both spring break and finals week of a semester that took place almost entirely online due to the COVID-19 pandemic. Some saw their interactions with the robot kitten as a nice change of pace and a welcome distraction (e.g., “It’s kind of like an interactive distraction I kind of like that, honestly. Having an interactive distraction is just kind of fun. It just never ends. In case you need something to do” and “It destresses your system of all the things you have to do because your brain [is] so jumbled up with, like, a whole bunch of tasks you’ve got to do… you’re like freaking out. This is not that. This is the absolute opposite of that”). One participant went on to elaborate on the importance of the robot’s ability to offer a distraction during such a stressful period of life:

One time, I specifically remember I was working on a big project that I had a due date coming up for and I was very, very stressed and I decided just on a whim as I was going back to the table where I work, I decided to grab that kitten robot to take with me. As I was typing I had it on my lap and it only meows for a little bit if you’re not petting it, so I tried to keep petting it, because it was comforting to have, have it with me as I was working, because I was very stressed (Interview 1).

The kitten was often a comforting distraction for participants as they faced both new and familiar stressors. The mental diversion of interacting with the kitten robot appeared to be enjoyable, even if that distraction was short-lived or minor (e.g., “Sometimes I thought it was kind of funny when it, like, moved its arms and stuff. So maybe a little bit there, I’d like laugh or something. But, I would not to say it [took] a huge load off my shoulders”).

##### 4.4.1.2 Positive Memories

In addition to the robot kitten offering a distraction, it also inspired participants to reminisce about positive memories that brought them comfort. For example, one participant noted, “It was also comforting when it would, um, purr. Because even though it was a robot, my interactions with real cats, like, I know that when they’re purring, that means that they’re content and it feels good.” This intentional recollection of how real cats respond enhanced the comfort this participant received from their interaction with the robot kitten. Other participants remembered their pets from home or childhood, and those positive memories influenced their perceptions as well:

It definitely reminded me of my cat at home and I think that was the main source of comfort from it, but after that, I, you know, once I got back home, I had my cat and it was kind of, it kind of pushed the robot cat out of the way because I had my real cat (Interview 6).

This participant reported finding comfort with the robot kitten because it triggered the memory of their cat from home. When reunited with their real cat, however, the robot kitten seemed insufficient as a replacement or source of comfort.

##### 4.4.1.3 Pleasure

Participants regularly commented on the enjoyment they received from tactile interaction with the robot kitten (e.g., petting, brushing, holding, etc.). Specifically, people noted how comforting it was to hold something so soft (e.g., “It did have a calming sensation when you cuddled it,” “I think a big part of that is, you know, tactile, it’s really soft and it’s, you know, relaxing to touch something soft,” and “whenever you hold something to your chest and, like, hold it, like, you always kind of get that feeling of just happiness and especially if it is warm and soft and, ya know, fluffy”). Simple, physical contact with the robot kitten seemed to help people relax and bring them comfort: “I think just, like, the fact that it’s like a little furry thing that you can pet. I feel like there’s just something about petting something that’s soft that kind of like, triggers that, like, comforting feeling.” Overall, participants enjoyed contact with the robot kitten because it was pleasing to touch and brought them comfort.

##### 4.4.1.4 Companionship

For some, the robot kitten was more than a stand-in for a real cat as it offered a genuine sense of companionship during a difficult time. In 2020 and 2021, the pandemic led to social isolation, and many institutions around the world enacted social distancing polices that included working remotely or conducting business/education online. The students in this study repeatedly mentioned COVID-19 as a source of stress and disruption to their lives. The robot kitten was a source of comfort by offering a predictable source of companionship:

Having this thing, it’s kinda nice, it’s kind of like having a personal friend to talk to you. It’s kind of nice. It also makes me kind of feel like I have an actual, like I have another friend I can talk to that is not an actual, that is not human. So, it’s kind of nice to have, it’s just kind of a nice thing to kind of have (Interview 2).

The lack of companionship due to social distancing and COVID-19 in this group of college-aged participants was difficult. They seemed to miss connecting with others and for some, the robot kitten filled that void, even if just for a short time (e.g., “Even when I’m playing a board game I have him sitting in my lap just kinda to have something at least, nonhuman entity to talk to”). The companionship offered by the robot kitten was available, but participants had to embrace that opportunity and for some, it just wasn’t sufficient (e.g., “I would not say it [was] comforting” and “I can’t say it would really help with my stress too much”).

##### 4.4.1.5 Summary

Almost all participants explained ways in which the robot kitten either facilitated or fell short in terms of contributing to feelings of comfort and wellbeing. Participants explained that, in terms of comfort, much depends on how the robot kitten fits into a person’s normal coping patterns and schedule (e.g., “I personally am glad that it went over finals week and that I had the kitten because if there is a potential for it to help people it’s a good time to give them something to help”; “To pet it, that was just comforting.”). Whereas a preference for solitude and quiet cuddles could readily accommodate the inclusion of the robot kitten, it made less sense to take it on social outings:

Most of my things that I’m stressed out about I usually…I like to go disc golfing with my roommates or something like that…whereas I guess if I were by myself and I didn’t have access to other people it [the robot kitten] would probably help a little bit.” (Interview 7).

In this way, results suggest that social support from companion robots will require fit with lifestyle and personality and that people may value either novelty or familiarity when seeking emotional benefits of these interpersonal outcomes.

Across the categories that emerged from our data set, we saw specific interpersonal outcomes that seemed to be based on aspects of imagined novelty, technology novelty, and relational novelty (Figure 2). Prior expectations, features, and behaviors of the device itself and the influence of other people all contributed to the experience of novelty for these participants. In other words, novelty was broader and more dynamic than just the newness of a piece of technology that fades after the initial interaction. Novelty is not something that confounds results or appears as unwanted noise within interactions. In fact, our study demonstrates that novelty is a multifaceted concept that needs to be unpacked based on one’s imagination, the technology itself, and the relational and temporal aspects of the experience (Figure 2).

## 5 Discussion

Through qualitative analysis of in-depth interviews with university students who used companion robot kittens in their homes for 6 weeks during the COVID-19 pandemic, we identified six themes relevant to understanding the experience of novelty in HRI. Holistically, our results show that novelty may be understood as a renewable resource, sensitive to socially interactive processes of re- and de-novelization, and existing in dialectical tension with familiarity/predictability. These insights may challenge prevailing assumptions that novelty is something that “runs out” or “wears off” permanently over time and that novelty declines in linear inverse proportion to familiarity.

Perhaps the most important finding to emerge from our results is the observation that, for our participants, novelty was not completely or permanently gone once people built initial familiarity with a social robot. In the beginning, their novelty experiences were tied to the new, exciting nature of the robot kitten itself. Participants first encountered novelty imaginatively, by forming expectations of the robot before receiving it, and then directly, by interacting with the robot after unboxing. Once feelings of familiarity and predictability settled in, the novelty/familiarity experience was more likely to arise through social interaction with others, when the robot proved valuable as a centerpiece of human conversation, and when other people’s reactions to the robot “rubbed off” on participants, either depleting or renewing their sense of novelty.

Understanding novelty as a renewable resource in HRI encourages moving beyond the idea that interaction patterns move only in one direction to recognize that people experience paradoxical needs and desires. Whereas a monologic approach to novelty and familiarity would frame them as either/or and a dualistic approach would view them as separate and unconnected experiences, a dialectical understanding of novelty/familiarity assumes that relationships are nonlinear, ever-changing, and characterized by contradictions that are worked through in interaction and communication (Baxter and Montgomery, 1996). The four core concepts of relational dialects are 1) contradictions, or the dynamic interplay of unified oppositions (e.g., novelty and familiarity), 2) totality, or the idea that a tension such as novelty and familiarity cannot be separated from other tensions like dependence and independence or privacy and openness, 3) process, or the notion that people fluctuate between the oppositions of a dialectic tension (e.g., moving between novelty and familiarity), and 4) praxis, or recognition of the role of individual choice and action in creating and recreating dialectical tensions (West et al., 2010).

As people’s interactions with robots become more commonplace, normalized, and sustained over time, there is the need to extend the scholarship of HRI deeper into the context of human-robot relationships (HRR). Doing so will require resourcing the available theoretical, analytical, and methodological tools for studying relationships that have emerged in social sciences and humanities disciplines. Social robots possess qualities that overlap not only with human actors involved in interpersonal and social processes but also with communication and media technologies. Therefore, understanding HRR will likely require melding and reconciling traditional psychology/interpersonal communication perspectives on relationship development, maintenance, and change with perspectives on people’s relationships to new media and technologies (e.g., parasocial relationships, mutual shaping of society and technology, domestication, diffusion). In the context of novelty, relational dialectics is a promising resource because it draws attention to how novelty and predictability tensions are experienced and navigated within all relationships and how the management of such tensions constitutes the relational culture between partners. Viewed from this angle, novelty is less a technocentric and fleeting feature of new devices and more a universal and tension-ridden experience endemic to the process of human relating.

Because people may handle the novelty/familiarity dialectic in various ways, it is important that designers recognize people’s ongoing and long-term needs for change, newness, and surprise as well as for stability, predictability, and familiarity, which will each be made meaningful only in relation to the other. Allowing people to successfully navigate interactions with social robots over time requires enabling them to toggle up novelty (for example, through updates, machine learning, programmable behaviors) when/if they wish. Whereas some participants stressed the importance of continued novelty experiences for long-term use and adoption, others appreciated the simplicity and privacy afforded by the robot kitten’s lack of connectivity to the internet or reliance on AI systems. These differences in situated needs for novelty reinforce the important role of designers in protecting users’ autonomy to determine when and to what degree novelty is re-introduced.

### 5.1 Measuring Novelty/Research Design Implications

In addition to the theoretical and design implications discussed above, there are also implications for research designs involving novelty in HRI. If novelty and familiarity/predictability are considered in a dialectical relation, then novelty is not a unidimensional feature of experience to be assessed independently of familiarity (Smedegaard, 2019). Both novelty and familiarity as well as their relation to one another as unified oppositions should be considered. Qualitative research designs will be particularly helpful in this regard because they allow participants to express in their own words how the tension between novelty and familiarity is felt, made sense of, and negotiated over time.

Based on our results, it seems unlikely there will be a set timeline or number of weeks when researchers say that “novelty effects have ended” because to the extent that people form relationships with robots, novelty effects (as a dialectic of praxis) will infuse the whole duration of life with the robot (HRR). This was evidenced by participants who said they will continue to hope and watch for the kitten robot to do something new and by the ongoing processes of socially interactive de- and re-novelization they described. Furthermore, the experience of novelty was not the same for all participants but was contingent on their views of the robot kitten, life circumstances, and ability to integrate the robot within their everyday activities, social circles, and identity needs. Therefore, rather than waiting x number of weeks or months to begin study of “real use or adoption,” assessing the subjective experiences of participants (their individual perceptions of novelty and familiarity, at the very least) within the research design would be more fitting.

### 5.2 Limitations and Future Directions

While this study provided some interesting analysis of novelty effects, it is not without limitations. First, the robot kittens were simplistic in their interactions and did not allow for more complex behaviors. Perhaps, as more commercial zoomorphic robots are developed, this study could be replicated with animal robots that allow for more complex interaction. There might be gaps between our initial expectations for interactions and the actual experiences of those using the robots (Lohse, 2011; de Graaf et al., 2018). However, as the analysis suggests, participants did find enjoyment and ways of interacting with these limited robot kittens. Additionally, the robots used in this study are relatively inexpensive when compared to other robots on the market. As such, with these kinds of robot animal designs, it might be possible to put more robots in the field in the hands of people who would benefit from this type of limited interaction. In doing so, it would be important for future studies to examine how people differentiate a robot from a toy and how various ontological comparisons may impact people’s impressions, uses, and experiences with technology.

Second, our intent was to give undergraduate students a robot kitten for interaction during COVID-19 restrictions in the United States. However, it is quite likely that these students experienced virtual schooling, social distancing, and overall issues related to the pandemic differently and may or may not have adhered to local and government policies. In other words, some participants might have kept going out to see friends and family despite health community guidelines and warnings. However, during the entire length of the study, the university participants attended only virtual classes and did not meet face-to-face. Further, our results may not speak to how typical these experiences are across populations, only that they are possible and interpretable. Future research needs to extend this study to socially isolated adult populations beyond those isolated due to the pandemic. Studies should also include various methods of data collection surrounding the actual interactions between people and the robot pets over time (e.g., diary studies, short video clips from participants, photos, observations, etc.). Finally, we do not suggest that our findings represent an exhaustive picture of novelty in HRI, but rather our results encourage us to keep exploring this topic and dig deeper into the complex nature of human-robot relationships.

### 5.3 Conclusion

In this study, we sought to better understand how people experience novelty and well-being with a robot kitten over 6 weeks. Across interviews, we found that participants encountered shifts in their interactions with the robot kitten that were tied to their own perceptions as well as encounters with others. In their own words, they talked about oscillating between familiarity with the robot kitten and a desire to be surprised by something new (i.e., novelty in dialectical tension with familiarity/predictability). Additionally, these findings might extend to adults living in relative social isolation despite the pandemic. As robot companion pets are marketed towards these adults, this study demonstrates how novelty effects might occur in the general population of social isolation. One’s ability to experience re-/de-novelization appeared to be a product of social encounters that occurred across the 6-week study, implying that the tension between novelty and familiarity was ongoing. Our findings suggest that novelty is not simply a confounding variable in HRI, but rather a dynamic resource tied to social engagement and individual preferences. This view challenges assumptions that novelty is a linear, constantly diminishing resource. In contrast, it appears that the complexity of novelty effects (as a dialectic of praxis) is an important consideration throughout the arc of human-robot relationships. Finally, we propose that novelty is both dynamic and complex, consisting of multiple components. In this study, we represent those different aspects of the experience as *imagined novelty, technology novelty*, and *relational novelty*. Each of these elements has an influence on the interpersonal experience of the user.

## Data Availability

The datasets presented in this study can be found in online repositories. The names of the repository/repositories and accession number(s) can be found below: https://osf.io/2qb34/?view_only=b7219fee2d89432bb26d89b433ff3f3c.
